# Erosive Potential of Pediatric Syrup Medications on the Human Enamel: Ex Vivo Study

**DOI:** 10.3390/dj13120588

**Published:** 2025-12-08

**Authors:** Fatima-Zohra Douiri, Amir Shayegan

**Affiliations:** 1 General Dentistry Department, Université Libre de Bruxelles, B-1020 Brussels, Belgium; zohradouiri@hotmail.com; 2 Children’s Hospital of Queen Fabiola, Université Libre de Bruxelles, B-1020 Brussels, Belgium

**Keywords:** pediatric syrups, enamel erosion, viscosity, acidity

## Abstract

**Background**: Pediatric syrups are frequently prescribed but may pose a risk to dental enamel due to their acidity and viscosity. Aim: To evaluate the erosive potential of commonly prescribed pediatric syrups on enamel from primary and permanent human teeth under ex vivo conditions. **Design**: Enamel–dentin blocks from sound primary and permanent teeth were assigned to nine groups (eight syrups and one control). Samples were immersed in their respective solutions four times daily for a 6-day exposure period. Mineral loss (ΔF) was assessed via Quantitative Light-Induced Fluorescence (QLF), surface roughness via profilometry, and morphological changes via scanning electron microscopy (SEM). Syrup pH and viscosity were also measured. **Results**: Significant ΔF changes were found only for dextromethorphan on primary enamel (*p* = 0.0054). No significant enamel loss was observed by profilometry. Surface roughness increased significantly with glycerin, distilled water, and azithromycin. Syrups showed a wide pH range (3.92–8.44) and varied viscosity, with ibuprofen and glycerin being the most viscous. **Conclusions**: Most pediatric syrups did not cause significant enamel demineralization or loss under short-term ex vivo exposure. However, increased surface roughness suggests that specific formulations may affect enamel texture, underscoring the need for preventive care in frequent users.

## 1. Introduction

Oral health is fundamental to a child’s growth, development, and overall well-being, serving as a cornerstone for overall health and quality of life. Among the various oral health problems, dental caries is the most common oral disease, recognized as one of the most prevalent infectious diseases worldwide [1]. Several studies have been conducted to explain its high prevalence, emphasizing the role of acids produced by bacterial fermentation of dietary carbohydrates, which gradually erode tooth structure [2].

Dental erosion is characterized by the progressive destruction of the hard tissues of the teeth caused by chemical processes. It results in the gradual loss of dental hard tissues through chemical dissolution, without bacterial involvement, and often occurs painlessly [3]. The acids responsible may be of intrinsic or extrinsic origin. However, in 1985, Smith and Knight suggested that intrinsic factors leading to tooth erosion may be involved in about a quarter of cases [4].

Exogenous causes of erosion are grouped into four major categories: environmental factors (e.g., exposure to sulfuric and nitric acids among dynamite factory workers), dietary factors (including acidic foods and beverage ingredients), behavioral factors (such as bruxism), and medicinal factors.

Pharmaceutical factors play a significant role in dental erosion, as many drugs contain acidic derivatives essential to their formulation. These acids are often necessary for preparing and preserving drugs, ensuring chemical stability throughout their shelf life. They help maintain the efficacy of the drug by preventing degradation, ensuring it remains physiologically compatible with the body, and improving its taste to enhance patient compliance [5]. However, frequent or prolonged exposure to these acidic components, particularly in liquid or chewable medications, can lower the mouth’s pH, contributing to the progressive demineralization of dental enamel and increasing the risk of erosion. In addition to acidic components, other factors such as frequent ingestion (i.e., at least twice a day), consumption at bedtime and between meals, high viscosity, and side effects like reduced salivary flow can contribute to the increased risk of dental erosion induced by medication intake [4,6,7].

Children generally prefer to take medicines in liquid form, such as syrups, rather than tablets or pills due to their sweet and pleasant taste. However, these sweetened and sometimes acidic syrups can harm oral health [8]. Given their widespread use in pediatric medicine, often for extended periods in the case of chronic illnesses, it is essential to investigate their potential impact on dental hard tissues [9].

Several studies have reported that liquid medications can reduce enamel hardness and induce morphological changes in the tooth surface [6,10]. However, the susceptibility of teeth to such damage may vary depending on whether the teeth are primary or permanent. Primary teeth differ from permanent teeth in several structural aspects: thinner enamel and dentin layers, lower mineral content, and a more permeable enamel surface. These characteristics make primary teeth inherently more vulnerable to acid-induced erosion and demineralization. Therefore, it is imperative to distinguish between the effects of medicinal syrups on primary and permanent enamel when assessing potential risks.

In this context, our study aims to evaluate ex vivo the effects of pediatric medicinal syrups available in pharmacies on the surface properties of enamel from both primary and permanent teeth. To comprehensively assess changes in enamel integrity, we employed several analytical techniques, including Quantitative Light-Induced Fluorescence (QLF), surface profilometry, and scanning electron microscopy (SEM).

The null hypothesis of this study is that these syrups do not significantly change enamel hardness or surface morphology.

## 2. Materials and Methods

Extracted human teeth (primary and permanent) were obtained from patients undergoing therapeutic or orthodontic extractions at the Children’s Hospital of Queen Fabiola, Brussels. Written informed consent was obtained from the parents or legal guardians, and this study protocol was approved by the Ethics Committee (CEH 51/14) date: 19 December 2024.

This ex vivo study was designed to evaluate the erosive potential of pediatric syrup medications on the enamel of extracted human teeth, both primary and permanent.

### 2.1. Materials

Nine pediatric oral formulations were selected based on their frequent prescription in pediatric practice: clindamycin, amoxicillin, azithromycin, dextromethorphan hydrobromide, acetaminophen, ibuprofen (sugar-free), sodium valproate, glycerol, and distilled water as the control solution. The active pharmaceutical ingredient (API), excipients, and physicochemical properties of each syrup are summarized in Table 1. In addition to the manufacturer-reported composition, the table includes the experimentally determined pH (measured by pH meter and titration), titratable acidity (mmol/L NaOH required to reach neutrality), viscosity (mPa·s), and major sweeteners/acidifiers. These physicochemical values were experimentally obtained in this study, with detailed results and graphical representations provided in the Results Section 3.

We selected permanent and primary teeth free from caries, white spots, or surface irregularities. The crowns of each tooth were methodically separated from the roots using a cylindrical diamond bur on a turbine. One hundred and seven enamel–dentin blocks were prepared and fixed to a solid support on the dentin side using Duraly resin. Enamel–dentin blocks were prepared from the vestibular surfaces of incisors and from the vestibular and lingual/palatal surfaces of primary molars and premolars, depending on the most intact and flat enamel regions available. These surfaces were selected to ensure standardization and suitability for QLF and profilometry analyses. The samples were divided into nine experimental groups and stored in distilled water until use.

The 107 specimens were randomly allocated into nine groups (n = 10 each, 5 primary and 5 permanent enamel blocks per group): eight groups exposed to pediatric syrups and one control group treated with distilled water (dH_2_O).

Distilled water was chosen as a negative control because of its neutral pH and absence of buffering ions, which makes it an accepted baseline in erosion studies.

Each enamel block was immersed in its allocated solution for 1 min, four times per day, over six consecutive days, simulating the average duration and frequency of pediatric drug intake. After each immersion cycle, the samples were rinsed, gently dried, and stored in fresh distilled water to avoid cumulative deposition.

The six-day exposure period was chosen to simulate a typical short-term course of pediatric medication, as most antibiotic and cough syrup treatments prescribed for children last approximately 5 to 7 days.

### 2.2. Quantitative Light-Induced Fluorescence (QLF) Test

**Figure 1:**
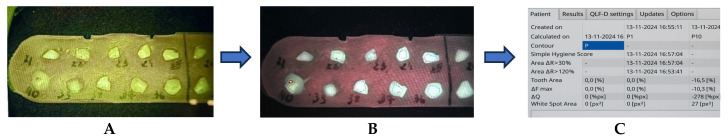
(**A**) Samples were numbered based on the pediatric syrup applied. The QLF device captured this baseline image under standard lighting before processing. (**B**) Fluorescent image from the QLF system highlighting demineralized areas. Outlined regions were used to calculate ΔF average, indicating fluorescence loss due to demineralization. (**C**) Data table showing ΔF average values (%) for each sample, used to assess demineralization caused by pediatric syrups.

The erosive effects of pediatric syrups on enamel were evaluated using Quantitative Light-Induced Fluorescence (QLF), developed by Inspektor Research Systems BV in Amsterdam, The Netherlands. This device utilizes high-intensity blue light to excite tooth tissue and captures the resulting autofluorescence. Demineralized enamel shows reduced fluorescence compared to healthy enamel. Digital images were taken at baseline (T0) and after exposure (T1). The specialized analysis software quantified the following: lesion area (in mm^2^) and lesion depth, expressed as the percentage of fluorescence loss (ΔF, %). A more negative ΔF value indicates greater mineral loss, suggesting a more significant erosive effect of the tested syrups (Figure 1).

### 2.3. Surface Profilometry

Surface topography was analyzed using 3D profilometry (Keyence VR-6000, Osaka, Japan). Measurements included surface roughness (Sa, Sz), volume change, and surface height differences. Baseline (T0) and post-exposure (T1) scans were compared.

### 2.4. pH Measurement Techniques

The pH of each pediatric syrup and of the control solution (distilled water) was measured using two complementary approaches.

#### 2.4.1. Direct Measurement

pH was recorded at room temperature using a calibrated digital pH meter (Milwaukee MW150, Milwaukee Electronics Kft, Szeged, Hungary). Three readings were taken for each sample, and the mean value was reported.

#### 2.4.2. Acid–Base Titration

To determine the buffering capacity, each sample was titrated with standardized NaOH (0.1 mol/L) under continuous stirring, and the volume required to reach pH 7.0 was recorded. Results were expressed as titratable acidity in mmol/L NaOH per liter of syrup. For formulations with initial pH values above 7.0, no NaOH was required (titratable acidity = 0). In these cases, titratable alkalinity was additionally determined by titration with standardized HCl (0.1 mol/L) until pH 7.0, expressed as mmol/L HCl per liter of syrup.

### 2.5. Viscosity Measurement

The viscosity of the nine test solutions was measured using a Ubbelohde-type capillary viscometer. All samples were equilibrated at 20 ± 0.5 °C before measurement as viscosity is temperature-dependent. Each solution was drawn above the upper timing mark by suction and allowed to flow freely under gravity through the capillary.

Dynamic viscosity (η, mPa·s) was calculated according to

η = K⋅ρ⋅t

where K is the viscometer constant, *ρ* is the solution density (g/cm^3^), and *t* is the flow time (s).

Each sample was measured in triplicate, and mean values were reported. For highly viscous formulations outside the measuring range of the Ubbelohde device (e.g., ibuprofen syrup), viscosity was determined using a rotational viscometer.

After each run, the viscometer was rinsed with molecular-grade water followed by acetone to ensure drying and to prevent cross-contamination.

**Scanning electron microscopic (SEM) analysis**

**Figure 2:**
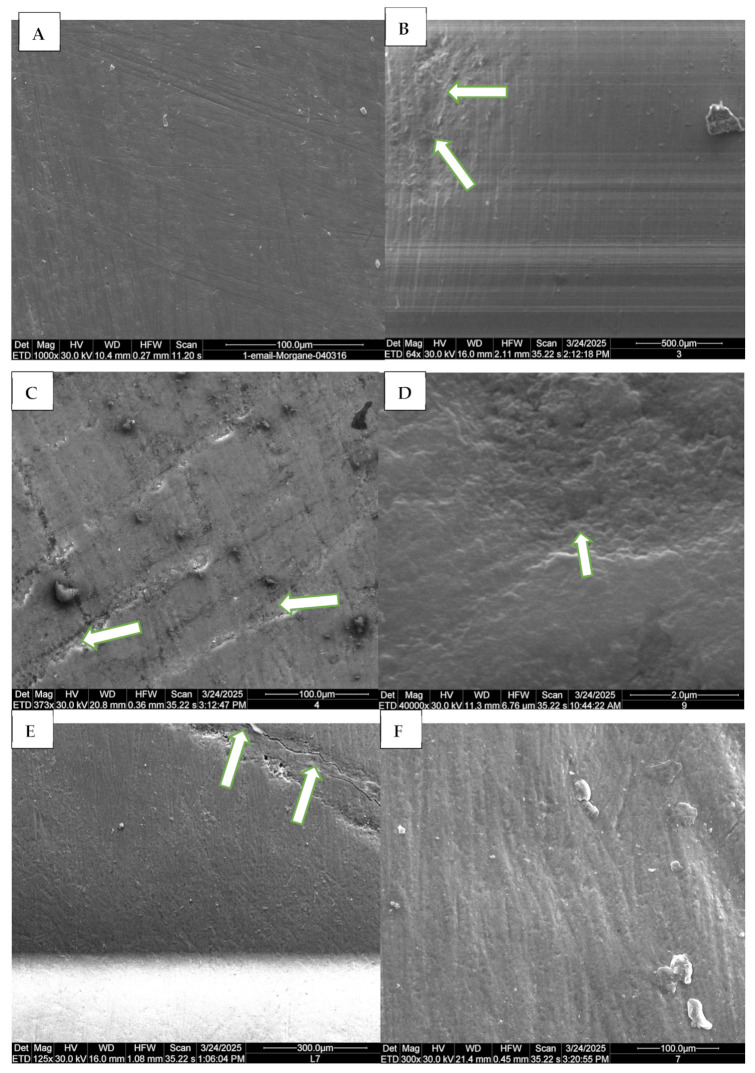
Representative SEM micrographs of dental enamel showing the erosive effects of pediatric syrups on primary and permanent teeth. (**A**) Control group, permanent tooth: intact enamel surface with no visible alterations. (**B**) Group 3 (Azithromycin, permanent tooth): presence of microcavities on the enamel surface (arrows), indicating the onset of surface degradation. (**C**) Group 4 (Dextromethorphan bromide, permanent tooth): small, scattered cavities associated with a fibrous appearance within the cavities (arrows), suggesting localized superficial demineralization. (**D**) Group 9 (dH_2_O, primary tooth): visible microcavities on the enamel surface, indicated by arrow. (**E**) Group 7 (Ibuprofen, primary tooth): microcavities with open and disorganized enamel prisms (arrows). (**F**) Group 7 (Ibuprofen, permanent tooth): pronounced fibrous appearance of the exposed enamel surface, indicating structural alteration.

Scanning electron microscopy (SEM) was used to qualitatively assess enamel surface morphology. Two enamel–dentin blocks per group were imaged at baseline (T0, untreated) and after six days of exposure (T1). Samples were dehydrated, vacuum-dried, and coated with a thin conductive layer of platinum to minimize surface charging. They were then analyzed under the SEM to capture detailed images of the surface (Figure 2).

### 2.6. Statistical Analysis

All data (ΔF values from QLF, profilometry parameters, pH, titratable acidity/alkalinity, and viscosity) were entered into Excel and analyzed with GraphPad Prism v.10.1.4 (GraphPad Software, La Jolla, CA, USA). Because the sample size in each subgroup was small (n = 5) and the normality tests have limited power under such conditions, a non-parametric approach was adopted throughout the study. Therefore, descriptive data are presented as the median and interquartile range (IQR) rather than the mean ± SD.

- Within-group comparisons (T0 vs. T1) were performed using Wilcoxon signed-rank tests.
- Comparisons among syrups were carried out with the Kruskal–Wallis test followed by Dunn’s post hoc test.
- Comparisons between primary and permanent teeth were performed using the Mann–Whitney U test.
- Correlations between physicochemical parameters (pH, titratable acidity/alkalinity, viscosity) and enamel outcomes (ΔF, Sa, Sz) were explored using Spearman’s rank correlation.

A *p*-value < 0.05 was considered statistically significant.

## 3. Results

### 3.1. Quantitative Light-Induced Fluorescence (QLF)

Analysis of QLF measurements revealed that, in most groups, enamel fluorescence loss (ΔF) did not differ significantly between baseline (T0) and post-exposure (T1), as assessed by the Wilcoxon signed-rank test.

The only notable change was observed in the Dextromethorphan Hydrobromide group (primary teeth), which showed a trend toward more negative ΔF values after exposure (*p* = 0.056), indicating a possible increase in enamel demineralization.

For all other syrups and for the control (dH_2_O), no statistically significant ΔF changes were detected in either primary or permanent teeth (Table 2). Likewise, intergroup comparisons using the Kruskal–Wallis test did not reveal significant differences.

Overall, these results suggest that, under the tested ex vivo conditions, the syrups generally did not cause measurable enamel demineralization detectable by QLF. However, Dextromethorphan showed a weak tendency toward greater fluorescence loss.

### 3.2. Profilometry Analysis

Profilometric analysis of surface roughness parameters (Sa and Sz) revealed similar overall patterns for both primary and permanent teeth (Table 3 and Table 4).

Data expressed as median (IQR) and analyzed using the Wilcoxon signed-rank test showed that, although most syrups produced slight increases in Sa and Sz values after exposure, these changes were not statistically significant.

In primary teeth (Table 3), a tendency toward higher roughness was observed for several syrups, including Glycerin, Azithromycin, Sodium Valproate, and Clindamycin (*p* ≈ 0.06–0.09). The control group (dH_2_O) also exhibited a similar trend for both Sa and Sz parameters.

In permanent teeth (Table 4), Sa and Sz values followed a comparable pattern, with slight post-exposure increases in most groups. Weak trends (*p* ≈ 0.06) were noted for Ibuprofen, Amoxicillin, Acetaminophen, and dH_2_O, although these differences did not reach statistical significance.

Overall, the profilometric findings indicate that exposure to the tested pediatric syrups led to minor increases in surface roughness, without significant enamel degradation, although some formulations showed borderline changes suggestive of a mild erosive potential.

### 3.3. pH Measurement

**Figure 3:**
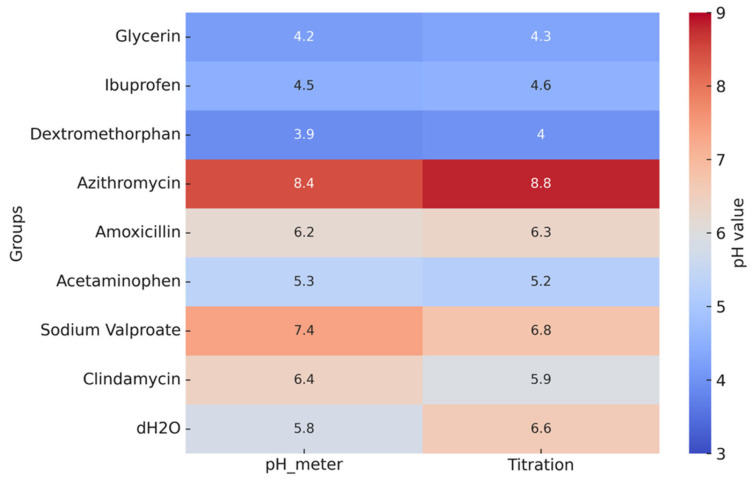
Heatmap of pH values of pediatric syrups measured by pH meter and titration. Colors represent pH values, with blue indicating lower pH and red indicating higher pH levels.

The pH values of the pediatric syrups, determined by both direct pH meter readings and titration, are shown in Figure 3. Dextromethorphan had the lowest pH (3.9–4.0), followed by glycerin (4.2–4.3) and ibuprofen (4.5–4.6). All these values fall below the critical enamel demineralization threshold (≈5.5), indicating a high erosive potential. In contrast, azithromycin exhibited the highest pH (8.4–8.8), making it clearly alkaline and unlikely to promote erosion under the tested conditions. Amoxicillin (≈6.2), acetaminophen (5.2–5.3), clindamycin (5.9–6.4), and sodium valproate (6.8–7.4) presented values close to neutrality, corresponding to a lower risk of erosion.

### 3.4. Viscosity Findings

**Figure 4:**
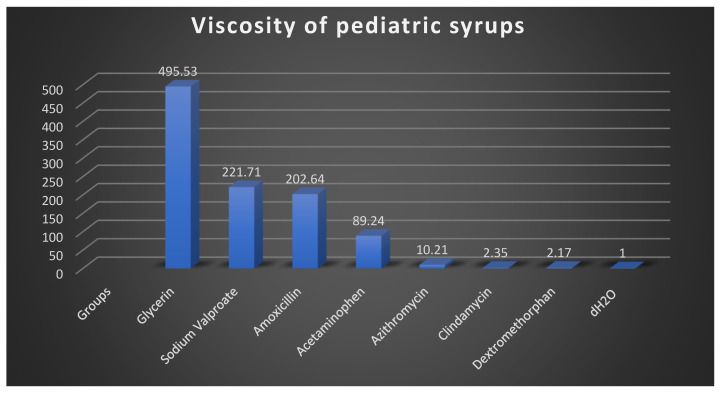
Viscosity measurements of the syrup solutions used in this study. Values represent the dynamic viscosity (in mPa·s) of each solution (mPa: millipascal; s: second). The viscosity of the ibuprofen syrup was too high to be measured using the Ubbelohde viscometer; a rotational viscometer is recommended for such high-viscosity samples. Ibuprofen syrup is therefore excluded from the viscosity data presented.

The viscosity values of the pediatric syrups are summarized in Figure 4. Ibuprofen exhibited by far the highest viscosity (76,583.73 mPa·s), although this value should be interpreted with caution, as the measurement required an extended time (≈2 weeks) and may have introduced bias due to the limitations of the Ubbelohde viscometer for highly viscous samples. Among the other formulations, glycerin showed the highest viscosity (495.53 mPa·s), followed by sodium valproate (221.71 mPa·s), amoxicillin (202.64 mPa·s), and acetaminophen (89.24 mPa·s). Azithromycin exhibited a relatively low viscosity (10.21 mPa·s), while dextromethorphan (2.17 mPa·s) and clindamycin (2.35 mPa·s) were close to the control (distilled water, 1 mPa·s).

Correlation analysis (Pearson’s test) showed weak, non-significant correlations for all comparisons (r ranging from −0.18 to 0.28, *p* > 0.05), indicating no clear association between pH, viscosity, and enamel changes under short-term ex vivo conditions.

## 4. Discussion

The present ex vivo study examined the erosive potential of commonly prescribed pediatric syrups on primary and permanent enamel. The evaluation utilized quantitative light-induced fluorescence (QLF), profilometry, pH measurements, and viscosity analysis over a 6-day exposure period. Liquid medications are widely used in pediatrics because of children’s difficulty swallowing solid dosage forms; however, excipients such as sugars, acids, and flavoring agents, rather than the active pharmaceutical ingredients, are recognized risk factors for dental erosion and caries [6,11]. This study, therefore, emphasizes the relevance of commercial formulations and their excipient profiles in determining erosive potential.

QLF analysis revealed no statistically significant changes in enamel fluorescence for most syrups, except dextromethorphan in primary teeth, which showed a reduction in ΔF. This finding is consistent with its low pH (~3.9) and highlights the higher susceptibility of primary enamel. However, the absence of a consistent pattern across other syrups suggests that short-term exposure may remain below the detection threshold of QLF for early demineralization. As noted in previous work, longer exposure times or repeated acid challenges are required to observe reproducible fluorescence loss [12].

Although Quantitative Light-induced Fluorescence (QLF) is a valuable non-destructive method for detecting early demineralization, its clinical application has several limitations [13]. These include the effects of plaque, staining, and surface irregularities, which can lead to an overestimation or underestimation of mineral loss. Ex vivo, under controlled conditions, as demonstrated in this study, QLF produces reliable comparative data. However, the results should be interpreted with caution when applied to clinical settings.

Despite the acidic pH of some syrups (e.g., glycerin, ibuprofen), the lack of significant ΔF changes in most groups suggests that short-term exposure alone may not be sufficient to induce substantial enamel demineralization detectable by QLF. Since QLF primarily detects subsurface mineral loss, a longer exposure duration or repeated acid challenges may be necessary to observe consistent effects. An in vitro study showed that QLF could detect enamel demineralization after 10 min of erosive challenge, with significant changes reported only after several intervals (20, 40, 50, and 60 min) [12].

Profilometric analysis supported these findings by showing no significant surface loss across groups. Minor variability in roughness parameters (Sa, Sz) within groups likely reflects initial sample heterogeneity or superficial surface changes rather than actual volumetric enamel loss. Distilled water was used as the control solution, consistent with previous erosion studies that routinely use it as a negative baseline [14,15,16,17]. Its neutral pH and lack of ions make it an appropriate reference, as it does not contain sugars, acids, or remineralizing agents. However, profilometry in our study revealed significant increases in Sa and Sz even in the distilled water group. Similar observations have been reported previously, as distilled water can affect enamel’s microstructure due to its hypotonic nature and lack of calcium and phosphate, which may promote a net outward flux of minerals during repeated immersion cycles [14]. While such effects are generally considered minimal compared with acidic challenges, their detection by profilometry underscores the sensitivity of surface roughness analysis to subtle topographic alterations. In contrast, QLF did not show significant changes with distilled water, suggesting that these modifications reflect superficial surface phenomena rather than subsurface mineral loss. These observations highlight that distilled water, though standard as a negative control, is not completely inert under ex vivo conditions, and its influence should be considered when interpreting profilometric outcomes.

Physicochemical measurements have highlighted the erosive potential of certain syrups [18,19]. Several products, such as dextromethorphan, glycerin, and ibuprofen, showed acidic pH values below the critical threshold for enamel dissolution (pH 5.5), indicating their ability to demineralize enamel hydroxyapatite crystals. In contrast, azithromycin exhibited an alkaline pH (greater than 8). However, subtle changes in surface roughness suggest that factors beyond pH, such as sweeteners, preservatives, and acidifiers, may also influence enamel interactions.

Viscosity is another key factor influencing erosive risk [20,21]. Highly viscous syrups, such as those containing ibuprofen and glycerin, exhibited the highest dynamic viscosity values, which may prolong their contact with the enamel surface. This prolonged retention could theoretically enhance acid exposure and reduce the effectiveness of salivary clearance ex vivo. In the present study, however, no direct correlation was observed between viscosity and enamel loss, possibly due to the relatively short exposure period and the static nature of the experimental setup. Unfortunately, due to limitations in measurement instruments, we could not quantify the viscosity of ibuprofen using the Ubbelohde viscometer, so it was not included in our statistical comparisons. Future research should utilize rotational viscometry for highly viscous formulations.

Our findings suggest that primary teeth may be more sensitive to acidic syrups, which aligns with previous research indicating that primary teeth have thinner enamel and lower mineral content. This increased susceptibility highlights the importance of implementing preventive strategies specifically designed for pediatric patients. However, the existing literature presents mixed outcomes. Some studies report significant erosion with prolonged exposure, while others emphasize the role of sugar content and dosing frequency in contributing to the risk of demineralization [9,22,23].

The current findings indicate that brief, short-term exposure may not result in significant enamel loss, particularly when protective oral factors like salivary buffering, pellicle formation, or mechanical abrasion are absent [12,13].

Notably, a differential response was observed between primary and permanent teeth, with primary enamel showing slightly greater sensitivity across specific assays. This pattern aligns with prior reports emphasizing the heightened vulnerability of primary dental tissues due to their thinner enamel structure and their frequent exposure to pediatric liquid medications [24].

The limitations of this study include its ex vivo design, the absence of saliva and biofilm, and the relatively short 6-day exposure period. The lack of salivary flow, buffering capacity, and biofilm formation in the experimental setup may underestimate the real erosive potential of pediatric syrups under clinical conditions, where saliva and the acquired pellicle play a crucial protective role against enamel dissolution. These factors limit our ability to extrapolate these findings to clinical settings. Another limitation of this study is the relatively small sample size per group, which may reduce statistical power. Future studies with larger sample sets are needed to confirm these findings. Despite these limitations, the data highlight potential risks associated with pediatric syrups that contain acidic and/or viscous excipients. Clinicians should advise caregivers on preventive measures, such as encouraging rinsing with water after medication intake, avoiding administration at bedtime, and opting for sugar-free or lower-viscosity alternatives when available. Future research should investigate long-term and repeated exposures under conditions that more closely mimic the oral environment, using outcomes such as microhardness, elemental analysis, and advanced imaging techniques.

Clinical Relevance: Pediatric dentists and healthcare providers should be aware of these risks and promote preventive practices such as rinsing with water post-medication, maintaining good oral hygiene, and considering sugar-free or alternative formulations when age-appropriate. Collaboration with pharmaceutical companies to improve formulations, such as adding remineralizing agents or reducing acidifiers, should also be encouraged.

## 5. Conclusions

This ex vivo study evaluated the erosive potential of commonly prescribed pediatric syrups on human primary and permanent teeth. The findings showed that most of the tested syrups did not cause significant enamel demineralization (ΔF) or surface loss (Sa, Sz) after short-term exposure, as measured by QLF and profilometry. Among the physicochemical parameters analyzed, pH and viscosity varied widely across products; however, correlation analysis revealed no significant relationships between these parameters and enamel changes.

These results indicate that short-term contact with pediatric syrups alone may not be sufficient to induce measurable erosion. However, factors such as low pH, high viscosity, prolonged exposure, and the absence of saliva in ex vivo conditions may influence outcomes. Future in vivo studies are warranted to confirm these findings under clinically relevant conditions and to assess the cumulative effect of repeated or long-term syrup use on enamel integrity.

## Figures and Tables

**Table 1:** Composition, pH, titratable acidity/alkalinity, viscosity, and excipient characteristics of the pediatric syrups tested.

Group	Syrup (Commercial Name)	Composition (API + Major Excipients)	pH (Meter)	pH (Titration)	Titratable Acidity (mmol/L NaOH)	Titratable Alkalinity (mmol/L Hcl)	Viscosity (mPa·s)	Sweeteners/Acidifiers
1	Clindamycin (Dalacin C 150 mg)	Clindamycin palmitate HCl, sucrose, …	5.80	5.92	83.3	0	2.35	Sucrose, cherry flavor
2	Amoxicillin (Clamoxyl 250 mg/5 mL)	Amoxicillin trihydrate, aspartame, maltodextrin, sodium benzoate, …	6.19	6.34	160	0	202.64	Aspartame, maltodextrin
3	Azithromycin (Zithromax 200 mg/5 mL)	Sucrose, trisodium phosphate, xanthan gum, glucose, flavors	8.44	8.82	0	21.5	10.21	Sucrose, glucose, citric acid traces
4	Dextromethorphan (Bronchosedal)	Glycerol, maltitol, citric acid, sodium citrate, …	3.92	4.03	12.5	0	2.17	Maltitol, citric acid
5	Glycerol (Balso Kids)	Dextromethorphan, sugar syrup, citric acid, …	4.19	4.30	7	0	495.53	Sugar, citric acid
6	Acetaminophen (Pediatric Dafalgan)	Acetaminophen, sugar, saccharin, citric acid, …	5.33	5.22	30	0	89.24	Sugar, saccharin, citric acid
7	Ibuprofen (Nurofen Pediatric, sugar free)	Ibuprofen, maltitol, saccharin, citric acid, …	4.52	4.56	11.8	0	N/A (too viscous for Ubbelohde)	Maltitol, saccharin, citric acid
8	Sodium valproate (Depakine 200 mg/mL)	Sodium valproate, saccharin, cellulose derivatives, …	7.37	6.77	0	40	221.71	Saccharin
9	Distilled water (dH_2_O, control)	-	5.8	6.60	0	0	1.00	-

**Table 2:** Summary of QLF results for enamel fluorescence loss (ΔF) before (T0) and after (T1) exposure to pediatric syrups. Data are expressed as median and interquartile range (IQR) for primary and permanent teeth (n = 5 each). *p*-values correspond to within-group comparisons using the Wilcoxon signed-rank test. ‡ 0.05 ≤ *p* < 0.10 (trend).

Group	Permanent Teeth T0 (IQR)	Permanent Teeth T1 (IQR)	Primary Teeth T0 (IQR)	Primary Teeth T1 (IQR)	*p*-Value Permanent Teeth	*p*-Value Primary Teeth
Glycerin	0.00 (0.00–0.00)	−0.50 (−1.10–0.10)	0.00 (0.00–0.00)	−1.10 (−1.60–0.40)	0.312	0.062 ^‡^
Ibuprofen	−6.50 (−6.90–0.00)	0.00 (−5.70–0.00)	0.00 (0.00–0.00)	0.00 (0.00–0.00)	0.587	0.479
Dextromethorphan	0.00 (0.00–0.00)	−2.20 (−5.10–0.70)	0.00 (0.00–0.00)	−5.40 (−6.20–5.30)	0.250	0.056 ^‡^
Azithromycin	0.00 (0.00–0.00)	−0.50 (−1.00–−0.10)	0.00 (0.00–0.00)	−1.60 (−2.90–0.80)	0.125	0.062 ^‡^
Amoxicillin	0.00 (0.00–0.00)	−0.50 (−0.90–−0.10)	0.00 (0.00–0.00)	−1.10 (−2.30–0.10)	0.125	0.187
Acetaminophen	0.00 (0.00–0.00)	−0.50 (−1.00–0.10)	0.00 (0.00–0.00)	−1.10 (−1.70–0.40)	0.500	0.125
Sodium valproate	0.00 (0.00–0.00)	−0.10 (−0.40–0.10)	0.00 (0.00–0.00)	−0.80 (−1.40–0.20)	0.125	0.062 ^‡^
Clindamycin	0.00 (0.00–0.00)	−0.40 (−0.70–0.00)	0.00 (0.00–0.00)	−1.00 (−1.50–0.40)	0.125	0.062 ^‡^
dH_2_O	0.00 (0.00–0.00)	0.00 (0.00–0.00)	0.00 (0.00–0.00)	0.00 (0.00–0.00)	1.000	1.000

**Table 3:** Rugosity parameters (Sa/Sz) changes in primary teeth pre- and post-syrup exposure. Data are expressed as median (interquartile range, IQR) (n = 5 per group). *p*-values were calculated using the Wilcoxon signed-rank test. ‡ 0.05 ≤ *p* < 0.10 (trend).

Group	Sa T0 (IQR)	Sa T1 (IQR)	*p*-Value	Sz T0 (IQR)	Sz T1 (IQR)	*p*-Value
Glycerin	73.80 (42.17–77.99)	58.85 (51.94–67.25)	0.187	423.50 (332.00–428.30)	423.50 (332.00–428.30)	0.062 ^‡^
Ibuprofen	54.96 (21.35–115.52)	79.47 (27.01–97.03)	0.312	302.50 (144.00–601.95)	402.20 (199.55–618.15)	0.312
Dextromethorphan	57.92 (27.21–89.21)	54.64 (43.78–96.80)	0.312	364.30 (249.15–566.55)	304.70 (285.20–545.25)	0.187
Azithromycin	54.49 (39.42–86.14)	71.84 (33.99–109.92)	0.062 ^‡^	377.20 (213.95–441.45)	386.10 (264.10–518.05)	0.187
Amoxicillin	75.28 (53.89–205.12)	71.25 (65.18–155.60)	0.312	417.70 (334.80–1164.30)	375.90 (364.50–798.35)	0.062 ^‡^
Acetaminophen	76.14 (43.15–92.24)	75.56 (30.65–94.87)	0.688	422.00 (246.60–492.50)	435.90 (201.75–520.30)	0.688
Sodium valproate	57.74 (35.26–64.88)	61.15 (29.43–80.65)	0.187	285.70 (202.80–358.60)	393.30 (168.40–410.50)	0.062 ^‡^
Clindamycin	76.62 (52.53–96.21)	82.51 (50.07–117.69)	0.187	452.10 (302.60–575.55)	420.10 (292.65–534.60)	0.125
dH_2_O	111.77 (85.57–120.56)	135.01 (92.48–147.34)	0.062 ^‡^	558.40 (442.85–636.00)	719.20 (456.05–789.80)	0.062 ^‡^

**Table 4:** Rugosity parameters (Sa/Sz) changes in permanent teeth pre- and post-syrup exposure. Data are expressed as median (interquartile range, IQR) (n = 5 per group). *p*-values were calculated using the Wilcoxon signed-rank test. ‡ 0.05 ≤ *p* < 0.10 (trend).

Group	Sa T0 (IQR)	Sa T1 (IQR)	*p*-Value	Sz T0 (IQR)	Sz T1 (IQR)	*p*-Value
Glycerin	74.21 (59.09–106.46)	85.98 (53.75–133.41)	0.062 ^‡^	456.10 (361.85–575.40)	480.90 (315.30–781.70)	0.062 ^‡^
Ibuprofen	71.56 (61.75–114.05)	89.00 (81.25–140.67)	0.062 ^‡^	455.40 (381.80–730.25)	577.20 (463.75–1532.45)	0.062 ^‡^
Dextromethorphan	72.15 (39.70–138.90)	85.56 (48.76–153.51)	0.125	347.10 (228.10–763.75)	447.20 (237.90–873.30)	0.062 ^‡^
Azithromycin	72.15 (66.20–97.78)	81.13 (44.37–99.91)	0.187	388.80 (340.60–539.30)	498.00 (276.80–567.85)	0.062 ^‡^
Amoxicillin	93.39 (61.44–145.13)	139.97 (75.09–153.87)	0.062 ^‡^	538.30 (450.80–819.45)	788.50 (591.65–907.65)	0.062 ^‡^
Acetaminophen	101.19 (86.73–146.68)	136.85 (106.01–196.35)	0.062 ^‡^	614.30 (487.15–869.95)	750.20 (609.30–1044.05)	0.062 ^‡^
Sodium valproate	75.70 (58.66–129.51)	84.25 (64.75–129.90)	0.187	431.70 (319.35–772.50)	586.30 (359.65–837.65)	0.062 ^‡^
Clindamycin	117.05 (95.59–200.10)	121.47 (92.42–221.73)	0.187	608.80 (511.20–962.65)	555.70 (481.10–1227.35)	0.062 ^‡^
dH_2_O	75.79 (66.42–108.32)	110.37 (81.85–118.19)	0.062 ^‡^	502.50 (387.65–632.65)	688.40 (466.65–757.85)	0.062 ^‡^

## Data Availability

All data generated and analyzed in this study are included in this published article. Additional datasets are available from the corresponding author on request.
